# Use of Nucleic Acid Analogs for the Study of Nucleic Acid Interactions

**DOI:** 10.4061/2011/967098

**Published:** 2011-07-24

**Authors:** Shu-ichi Nakano, Masayuki Fujii, Naoki Sugimoto

**Affiliations:** ^1^Faculty of Frontiers of Innovative Research in Science and Technology, Konan University, 7-1-20 Minatojima-Minamimachi, Chuo-ku, Kobe 650-0047, Japan; ^2^Frontier Institute for Biomolecular Engineering Research, Konan University, 7-1-20 Minatojima-Minamimachi, Chuo-ku, Kobe 650-0047, Japan; ^3^Department of Environmental and Biological Chemistry, Kinki University, 11-6 Kayanomori, Iizuka, Fukuoka 820-8555, Japan; ^4^Molecular Engineering Institute, Kinki University, 11-6 Kayanomori, Iizuka, Fukuoka 820-8555, Japan

## Abstract

Unnatural nucleosides have been explored to expand the properties and the applications of oligonucleotides. This paper briefly summarizes nucleic acid analogs in which the base is modified or replaced by an unnatural stacking group for the study of nucleic acid interactions. We also describe the nucleoside analogs of a base pair-mimic structure that we have examined. Although the base pair-mimic nucleosides possess a simplified stacking moiety of a phenyl or naphthyl group, they can be used as a structural analog of Watson-Crick base pairs. Remarkably, they can adopt two different conformations responding to their interaction energies, and one of them is the stacking conformation of the nonpolar aromatic group causing the site-selective flipping of the opposite base in a DNA double helix. The base pair-mimic nucleosides can be used to study the mechanism responsible for the base stacking and the flipping of bases out of a nucleic acid duplex.

## 1. Introduction

Nucleic acids have many remarkable properties that other molecules do not possess. The most notable property is the ability of sequence-specific hybridization through Watson-Crick base pairing. Even a short oligonucleotide sequence, readily synthesized chemically and available on the market at a relatively low cost, can self-assemble into a defined structure and hybridize specifically to a target sequence in accordance with the base pair-rule of A/T and G/C. Importantly, the controls of the self-assembly and the hybridization are not difficult when one considers the interaction energy of nucleic acid reactions [1]. Additionally, it is possible to conjugate with other molecules, such as fluorescent dyes, amino acids, and nanoparticles. Thus, the methodologies that utilize DNA and RNA oligonucleotides as a tool for technology such as nanomaterial and medicinal and therapeutic usages have become of broader interest over the past decades. 

**Figure 1:**
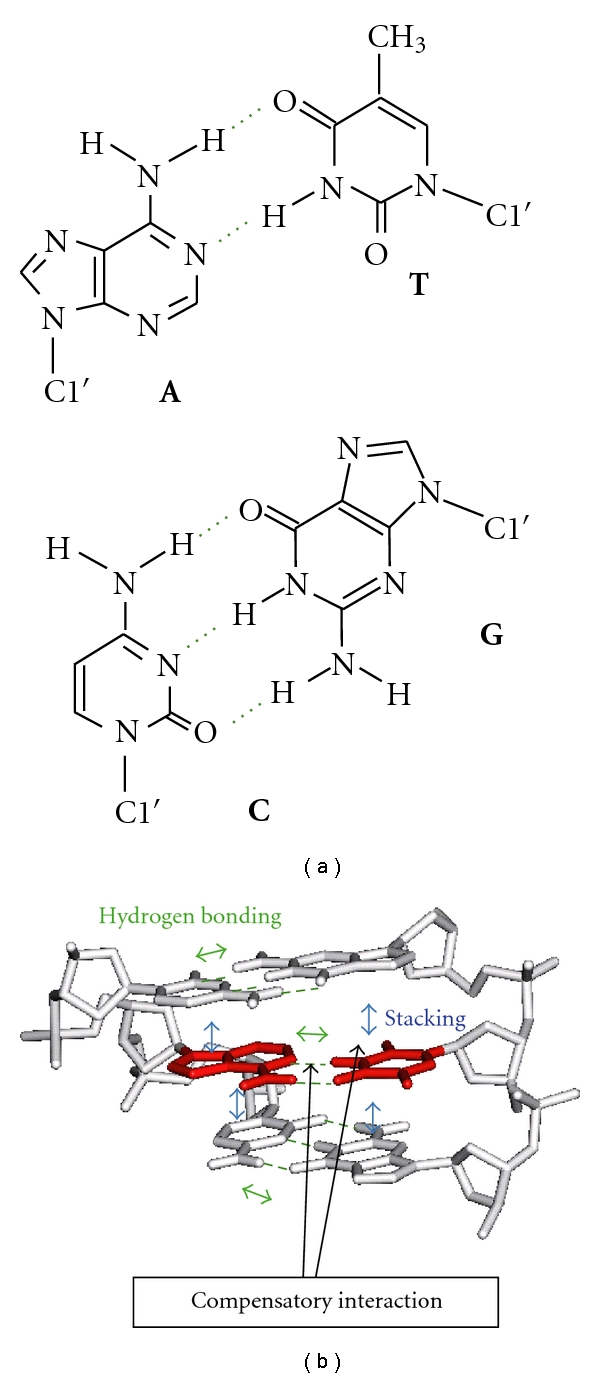
(a) Watson-Crick A/T and C/G base pairs. C1′ represents the 1′ carbon atom of deoxyribose in DNA. (b) Interbase hydrogen bonding and stacking interactions formed in a DNA duplex. A compensatory relationship is suggested between the interaction energies of the hydrogen bonding and the base stacking.

The most common structure formed by base pairing is the right-handed double helix. The geometry of Watson-Crick base pairs mediated by hydrogen bonding is similar regardless of the nucleotide sequence, and this allows a double helical conformation virtually identical without disrupting coplanar stacking between adjacent base pairs. Interbase hydrogen bonding is responsible for the association of complementary bases, which is essential for the storage and retrieval of genetic information. Hydrogen donors and acceptors on the purine and pyrimidine bases direct the base pair partner by forming two hydrogen bonds in the A/T pair and three in the C/G pair (Figure 1(a)). According to the number of hydrogen bonds, the C/G pair appears more stable than the A/T pair. However, because base stacking is formed simultaneously with the hydrogen bonding, both interactions contribute to the integrity and the thermodynamic stability of base-paired structures. In contrast to hydrogen bonding, the base stacking does not demand a particular interaction partner, while the interaction energy between purine bases is usually greater than that between pyrimidine bases due to the larger overlapping area of purine bases. The strength of the stacking interaction has particular relevance to the conformation of unpaired nucleotides, for example, single-stranded overhangs and the helical junction containing a nick site, whether stacked or bent [2–5]. The degree of stacking is also important for the design of fluorescent dye molecules attached to an oligonucleotide [6]. It is an important feature in nucleic acids that the base pair is formed in concert with the binding of cations and water molecules. Because the base pairing brings the sugar-phosphate backbones close to each other which increases the electrostatic repulsion between the phosphate groups, counterions must bind to nucleic acids through Coulomb interaction [7]. Formation of the base pairs also accompanies rearrangements of the hydration layer surrounding nucleic acid chains, especially around the bases and within the helical grooves [8, 9].

The nearest-neighbor model is widely used to account for the thermodynamic behavior of Watson-Crick duplexes. The model assumes that the base pair formation is mostly affected by adjacent (nearest-neighbor) base pairs by taking into account the contributions from base stacking as well as interbase hydrogen bonding. Nearest-neighbor parameters for base pairing have been extensively investigated, and the Gibbs free energy at 37°C (Δ*G*_37_°) that ranges from –0.2 to –3.4 kcal mol^−1^ (1 kcal = 4.18 kJ) for each nearest-neighbor base pair is useful to predict the hybridization energy and folding structures of DNA and RNA [2, 10]. Although the energy data include contributions from the hydrogen bonding and the base stacking, the free-energy increments from each interaction have been estimated from the studies using unnatural nucleotides and dangling end residues and by manipulating known loop interactions [11–13]. Interestingly, the quantitative data suggest that the free energies for forming a single hydrogen bond and the stacking interaction are comparable to each other, providing from –0.2 to –1.8 kcal mol^−1^ in Δ*G*_37_° under a competitive correlation (Figure 1(b)), where the base pairing with a lower hydrogen bond energy provides a greater stacking energy [11]. The phenomenon can be accounted for by assuming the interaction mechanism in which the geometry optimized for interbase hydrogen bonding is not suitable for base stacking and *vice versa*. On the other hand, investigations of the coaxial stacking of nicked and gapped sites suggest that base stacking is the major stabilizing factor in a double helical structure of DNA [3, 5]. Studies on the stacking interaction are important for understanding not only the fundamental aspects of nucleic acid interactions but also the biological processes involving base pair formation and strand opening, such as DNA replication and refolding of nucleic acid structures. 

Many unnatural nucleosides have been explored according to various demands of researchers. They have been modified or replaced the nucleotide base (*C*5-modified uridine nucleosides, *N*3-modified cytidine nucleosides, nonpolar nucleosides replaced with an aromatic hydrocarbon group, etc.) or the sugar-phosphate backbone (2′-*O*-modified RNA, phosphorothioate DNA, morpholino oligonucleotide, peptide nucleic acid, locked nucleic acid, etc.), as introduced in preceding articles (e.g., [14–16]). In this, we briefly introduce the nucleic acid analogs possessing an unnatural stacking group. We also describe the nucleoside derivatives of a base pair-mimic structure that we have examined to understand the biochemical properties of nucleic acid interactions, for example, the mechanisms responsible for the nucleotide base stacking and the flipping of bases out of a nucleic acid duplex.

## 2. Unnatural Nucleosides That Mimic Nucleotide Bases

**Figure 2:**
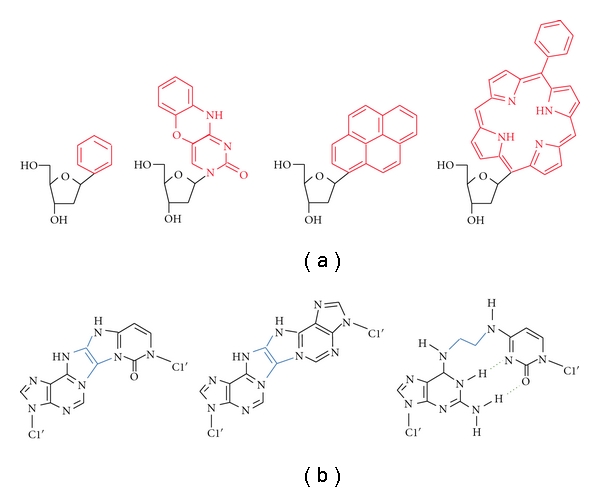
(a) Structures of unnatural nucleosides as a base analog with an aromatic hydrocarbon group in place of the purine and pyrimidine bases. (b) Structures of the base pair analogs that provide the interstrand crosslinking sites. The covalent bonds linking the nucleic acid bases are highlighted in blue.

There are many reports of unnatural nucleosides developed for various purposes. Some are aimed at enhancing the affinity and selectivity in targeting to DNA and RNA sequences by increasing the number of hydrogen bonding sites or by addition of extra aromatic rings to the pyrimidine base [14]. The DNA base analogs lacking particular hydrogen-bond donor and acceptor groups are also used to investigate the influences of the polar groups in DNA bases on the base pair stability [17]. On the other hand, many nonhydrogen-bonding analogs with an aromatic hydrocarbon group in place of the base have been explored (some examples are given in Figure 2(a)). Planar aromatic molecules of an expanded size are beneficial for increasing the interaction energy. If the aromatic group lacks the atoms involved in hydrogen bonding, they may pair with any of the natural bases with little discrimination [15, 18]. The nonpolar base mimics of an aromatic hydrocarbon group, such as benzene, naphthalene, and pyrene, attached to C1′ of ribose in place of the purine and pyrimidine bases were incorporated at the end of and in the center of a DNA strand [19]. It was found from the research that a less-polar compound stacked more strongly when molecules of the same size were compared and that the pyrene stacking was the strongest among the tested aromatic groups. However, it is known that the interaction with a strong stacking group often disrupts the helical structure of DNA. For example, planar polycyclic surrogates possessing fused 1–3 aromatic rings or more intercalate into a DNA duplex and perturbs the helix conformation [20–23]. The covalently appended quinoline residue at the terminal of an oligonucleotide also largely disrupts the DNA duplex structure [24]. The large aromatic groups of the pyrene-modified and porphyrin-modified nucleotides inserted into a DNA helix are found to interfere with the opposite base stacking and are forced to flip to an extrahelical position [25, 26]. The energy cost for the base flipping is quite high due to the loss of base stacking, but it can be compensated by intercalation of the large nonpolar aromatic group into the duplex.

Several types of compounds to introduce a covalently linked base pair portion have been developed to provide interstrand crosslinking sites in DNA strands (Figure 2(b)). In principle, the covalent bonds adducting between the probe strand and a target sequence are not dissociable, so that they are assumed to be useful for applications in gene regulation. There are two strategies for incorporating covalently linked sites in a DNA duplex. One is to use a fused base pair analog consisting of purine and pyrimidine nucleosides linked by covalent bonds [27, 28]. An alternative strategy is to use an unnatural nucleoside bearing a reaction group for alkylation, Schiff base formation, or other types of covalent bond formation triggered by the addition of a reaction reagent or the exposure to a light [29, 30]. Formation of the covalent bonds between two bases is triggered by a sequence-specific hybridization with a target sequence, while particular metal ions (e.g., Ag^+^, Hg^2+^, and Cu^2+^) can mediate covalent bonding with the use of natural bases as well as unnatural bases [31, 32]. Because the covalent bonds are formed only when a target site is located at a close distance, molecular design considering the distance between the crosslinking group and the target site is critical. 

Chemical synthesis using the solid phase method is widely used for site-selective incorporations of unnatural nucleosides by preparing their phosphoroamidite derivatives. On the other hand, DNA polymerase reaction can be applied, especially for incorporation at multiple sites and into a long DNA strand. Many pairs of base analogs that extend the genetic code have been reported [33, 34]. They are selectively incorporated into a DNA strand at desired positions using DNA polymerase, in accordance with their hydrogen-bond donor and acceptor sites and even through steric complementarity of the shape and size of the base analogs. Examinations of whether unnatural nucleosides can be used as a substrate for biological enzymes are important for applications as an anticancer drug and an agonist of receptors and enzymes [35, 36].

## 3. The Base Pair Analogs of a Base Pair-Mimic Structure

### 3.1. Design and Synthesis of the Base Pair Analogs Tethering a Nonpolar Stacking Group

**Figure 3:**
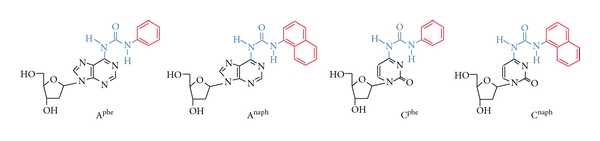
Structures of the base pair-mimic nucleosides of deoxyadenosine and deoxycytidine derivatives tethering the nonpolar aromatic group (colored in red) through an ureido linker (blue).

**Figure 4:**
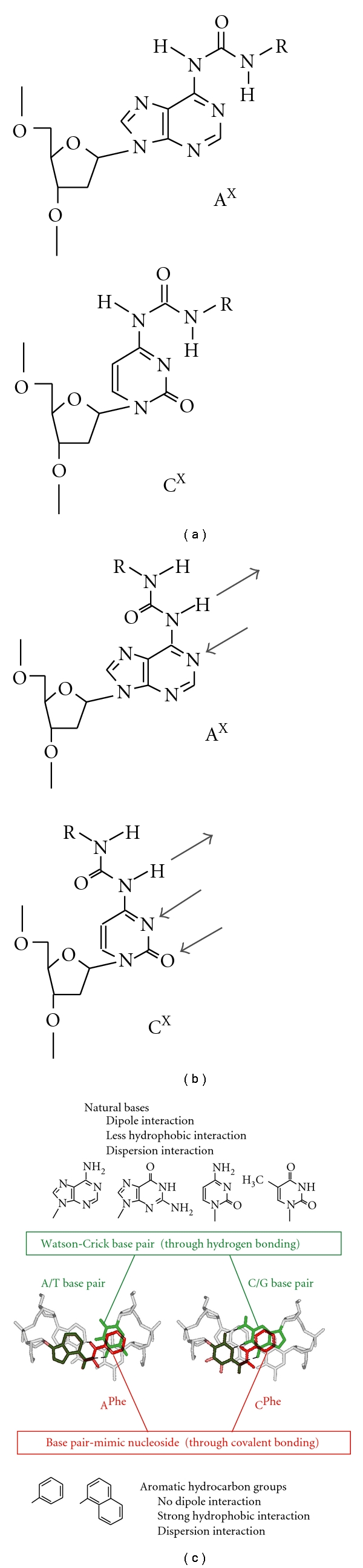
(a) and (b) Possible conformations of the deoxyadenosine and deoxycytidine derivatives, the nonpolar group stacking conformation (a) and the base pair conformation (b). indicates the phenyl or naphthyl group. The arrow indicates the site of hydrogen bonding with a complementary base. (c) Comparison of the major interaction forces for the stacking of the A/T and C/G base pairs and the stacking of the A^phe^ and C^phe^. The nonpolar aromatic group in the base pair-mimic nucleosides is indicated in red, and the complementary base is indicated in green.

Stacking interaction of the purine and pyrimidine bases is mediated by the combination of electrostatic, hydrophobic, and dispersive forces. Although the base pair interaction energy is well studied, the mechanism responsible for the base stacking is poorly understood. We are aiming to understand better the biochemical properties of nucleic acid interactions and the mechanisms behind the stacking interaction by using base pair analogs. For the interaction mechanism study, it is important to design nucleic acid analogs that are compatible with the interaction geometry of canonical base pairs in a double helical conformation. We had designed the compounds tethering a simple aromatic hydrocarbon group of a base pair-mimic structure, as shown in Figure 3: the deoxyadenosine derivatives containing the phenyl group A^phe^ (*N*6-(*N*′-phenylcarbamoyl)-2′-deoxyadenosine) or the naphthyl group A^naph^ (*N*6-(*N*′-naphthylcarbamoyl)-2′-deoxyadenosine) and the deoxycytidine derivatives containing the phenyl group C^phe^ (*N*6-(*N*′-phenylcarbamoyl)-2′-deoxycytidine) or the naphthyl group C^naph^ (*N*6-(*N*′-naphthylcarbamoyl)-2′-deoxycytidine). The base pair analogs of A^X^ and C^X^, where X is phe or naph, have a nonpolar base analog of the phenyl or naphthyl group attached to the amino group of deoxyadenosine or deoxycytidine by an ureido linker. Thus, the configuration of the ureido linker is associated with the orientation of the nonpolar aromatic group. The phenyl and naphthyl groups can stack with a nucleic acid duplex when adopting the base pair-mimic geometry, of which the nonpolar base analog occupies the Watson-Crick face of the adenine or cytidine moiety (Figure 4(a)). According to the molecular modeling study, the naphthyl group as well as the phenyl group can be accommodated in a DNA duplex without significant perturbation of the sugar-phosphate backbone conformation when the opposite nucleotide base is absent. On the other hand, the base pairing with a complementary base, A^X^/T and C^X^/G, through intermolecular hydrogen bonding is allowed when the nonpolar aromatic group is located out of the helix (Figure 4(b)). The potential to adopt two different conformations is characteristic of the base pair-mimic nucleosides shown in Figure 3. It is important to note that the stacking mechanism between the natural bases and the nonpolar aromatic groups is different (Figure 4(c)). In general, stacking of a planar aromatic group can be mediated by the combination of electrostatic, hydrophobic, and dispersive forces. However, less contributions from the hydrophobic effects are suggested for the stacking of natural bases, while the hydrophobic effect and dispersion become more significant than electrostatic forces for the stacking of nonpolar groups [37–40].

Chemical synthesis and incorporation of the base pair-mimic nucleosides into a DNA strand are simple. Synthesis of the deoxyadenosine and deoxycytidine derivatives can be started with 2′-deoxyadenosine and 2′-deoxycytidine, respectively (see the supplemental data in [41]). The compounds are incorporated into an oligonucleotide at high efficiency using an automated synthesizer based on phosphoroamidite chemistry. We have prepared the DNA oligonucleotides bearing A^X^ or C^X^ at the end of and in the middle of a sequence. The thermal melting curve was determined to obtain the thermodynamic parameters for DNA structure formations in the 1 M NaCl-phosphate buffer at pH 7.0, which is the condition widely used for determining the stability of oligonucleotide structures. The duplex conformation was investigated using circular dichroism (CD) spectra, a fluorescent base analog, and polyacrylamide gel electrophoresis.

### 3.2. Dangling End Stacking of the Base Pair-Mimic Nucleosides

**Figure 5:**
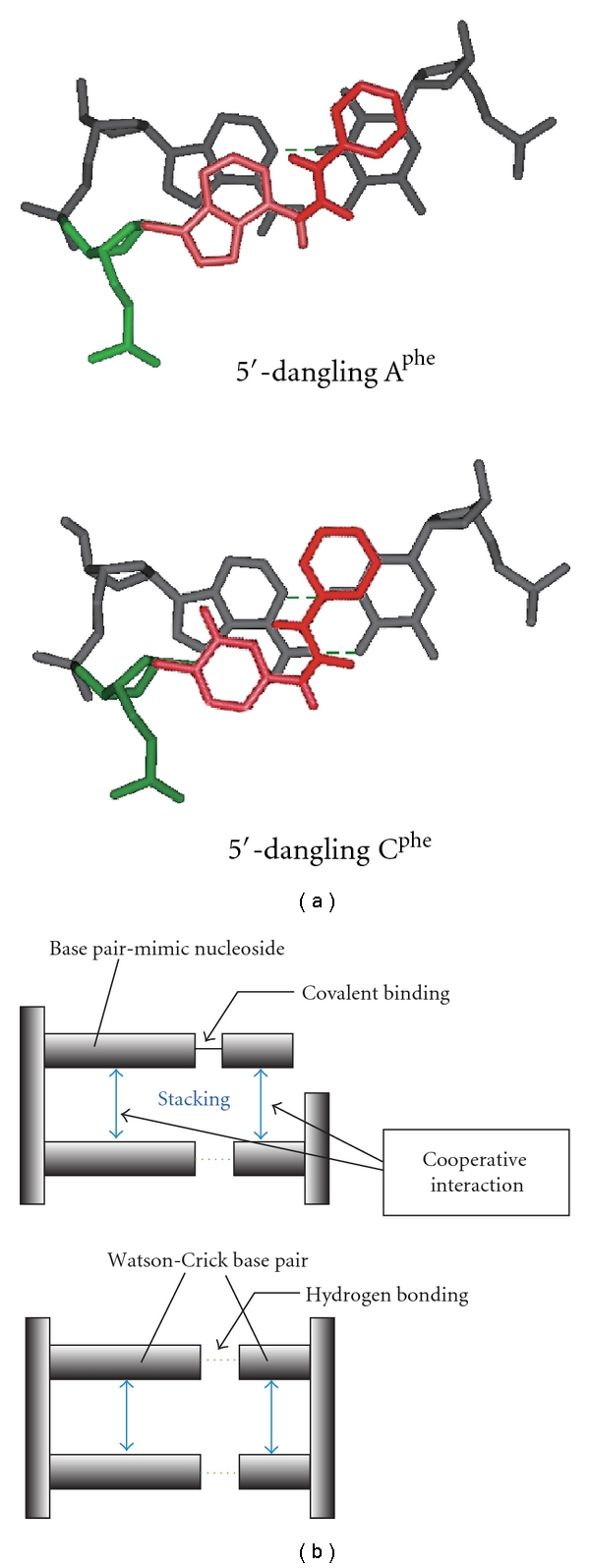
(a) Stacking conformations of the dangling A^phe^ and C^phe^ (colored in red) at the 5′-end of a DNA duplex. (b) Side view of the DNA double helix, representing the interaction mechanism of the dangling end stacking (upper) and the Watson-Crick base pairing (lower). Cooperative interaction in the base pair-mimic nucleoside is suggested between stacking of the base moiety and stacking of the nonpolar group.

According to the nearest-neighbor model, energy contribution from the stacking interaction can be evaluated from the interaction energy between the unpaired dangling residue and the adjacent base pair at a helix terminus [42, 43]. Duplex stability increases when the dangling end stacking is significant. For natural DNAs, increments in the interaction energy by a single dangling end ranges from 0.48 to −0.96 kcal mol^−1^ in Δ*G*_37_°, depending on the dangling end residue, its position at either 5′ or 3′ of the strand, and the adjacent base pair [42, 43]. Particularly, a dangling A increases the duplex stability by 0.1~−1.0 kcal mol^−1^, which is greater than that provided by a dangling C (0.3 ~−0.5 kcal mol^−1^), indicating the greater stacking strength of adenine than of cytosine. In contrast, we found that both A^X^ and C^X^ provided much more interaction energy (−0.1~−1.8 kcal mol^−1^) [44, 45], which was comparable to the formation of Watson-Crick A/T and C/G base pairs (–0.5~−1.8 kcal mol^−1^) and the stability reported for the dangling pyrene-modified nucleotide (−1.7 kcal mol^−1^) [19, 38]. The large stabilization energy suggests that the nonpolar aromatic groups efficiently stack with the terminal base pair by adopting the base pair-mimic geometry as indicated in Figure 5(a), in which the ureido linker may interact with *N*1 of adenine or *N*3 of cytosine.

The dangling end study provides valuable insights into the stacking energy contributed from the nonpolar aromatic groups. The stabilization energies from the dangling A^phe^ and A^naph^ were similar to each other, and those from C^phe^ and C^naph^ were as well. The similarity in the energy contributions from the phenyl group and the naphthyl group suggests that the overlapping area of the stacking group, which is relevant to the dispersive and hydrophobic contributions, is not the major determinant for the stacking energy. It has been proposed that the dominant contribution to the stabilization from a dangling end nucleotide comes from the stacking conformation that covers the atoms participating in the hydrogen bonding of an adjacent base pair [4]. In fact, the hydrogen-bonding atoms of the terminal base pair are well covered with the stacked phenyl and naphthyl groups and the ureido linker (Figure 5(a)). It is an interesting finding that large stabilization energy was provided by C^X^ regardless of the low stacking energy from the cytosine alone. This observation suggests a positively cooperative interaction between the stacking of the base moiety and the stacking of the nonpolar aromatic group. The interplay in the interactions of two planar aligned stacking groups could also be inherent in Watson-Crick base pairs that are noncovalently linked to each other (Figure 5(b)).

### 3.3. Mechanism of the Base Stacking Interaction Revealed by the Base Pair-Mimic Nucleosides

**Figure 6:**
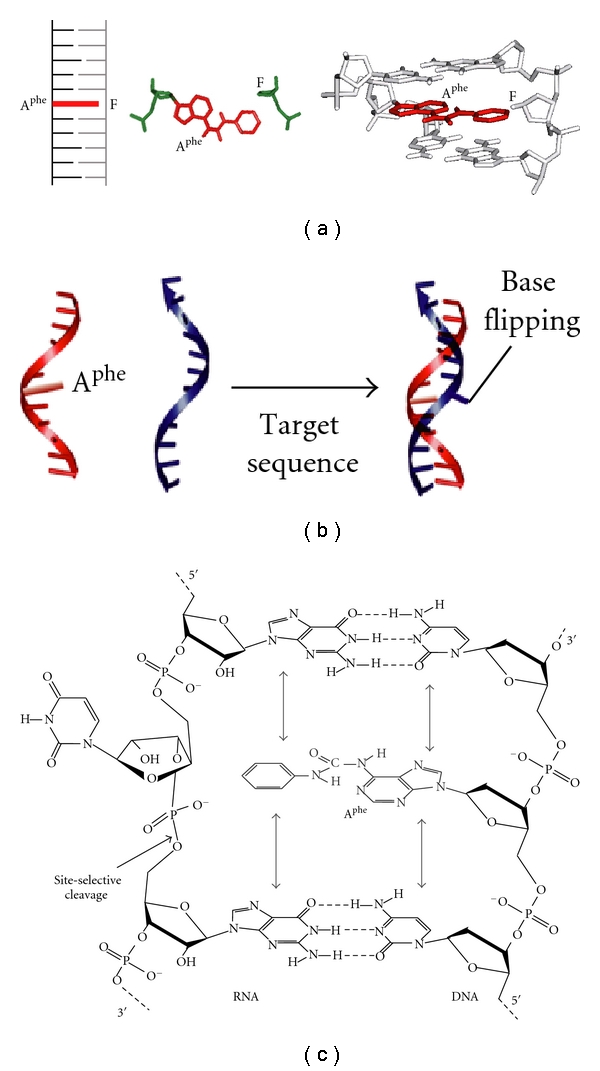
(a) The stacking conformation of A^phe^ opposite an abasic site F in the center of a DNA duplex. (b) Hybridization of the DNA strand bearing A^phe^ with a complementary target strand, followed by the formation of the flipped-out conformation. (c) The base flipping conformation induced by A^phe^ in an RNA/DNA duplex. The hybridized RNA strand is cleaved site-selectively at the 5′-side of the phosphodiester bond of the flipped-out ribonucleotide.

The stacking circumstances between at the terminal and in the center of a DNA duplex are quite different. There is more susceptibility to base pair fraying and water accessibility at the terminal than at the center of a DNA strand. Additionally, there is no remarkable conformational restriction for the stacking at the end; thus the stacking interaction by a dangling end residue can be maximized while the stacking geometry in the center of a DNA duplex is highly restricted. For comparison with the dangling end stacking, we further investigated the DNA duplexes bearing the base pair-mimic nucleosides in a helix center. Because severe steric hindrance with the opposite nucleotide base was expected, the DNA duplexes bearing tetrahydrofuran as an abasic site analog were investigated (Figure 6(a)) [41]. Introduction of the abasic site in an 11-mer DNA duplex largely decreased the duplex stability (by 5.2 kcal mol^−1^) due to losing the base stacking and providing additional flexibility to the helix. However, the duplex stability was markedly restored by the replacement of A by A^phe^ (−3.0 kcal mol^−1^, as the restored free energy) or A^naph^ (−4.1 kcal mol^−1^) opposite the abasic site and also by the displacement of C by C^phe^ (−2.7 kcal mol^−1^) or C^naph^ (−3.6 kcal mol^−1^). The thermodynamic data indicate intercalation of the nonpolar aromatic groups in the DNA duplex by adopting the base pair-mimic geometry presented in Figure 6(a). In contrast to the case of dangling end stacking, the interaction from the naphthyl group was stronger than that from the phenyl group, and their interaction energies were obviously lower than the formation of a Watson-Crick base pair. 

It is a remarkable finding that, although the interaction mechanism differs between the base pair-mimic nucleoside and the Watson-Crick base pair, a linear free-energy correlation between them are exhibited: as the interaction free energy from the base pair analog increases, the interaction energy for the corresponding base pair formation (A^X^/F for A/T base pair, and C^X^/F for C/G base pair) increases [45]. A similar relationship was obtained with the dangling end data relative to the corresponding Watson-Crick base pairs (A^X^ for A/T base pair and C^X^ for C/G base pair). These observations suggest that the major interaction mechanism that determines the strength of DNA base stacking is maintained in the aromatic group stacking, even though the phenyl and naphthyl groups lack significant dipole moments and hydrogen bonding sites. This finding is useful to understand nucleic acid interactions and to design new unnatural nucleotides with aromatic groups for use in diverse applications.

Our base pair analogs are also useful for the study of DNA hydration. Hydration of a DNA duplex has been extensively studied from the structural point of view [46–50]. While the purine and pyrimidine bases have the hydration sites of the amino group and oxygen and nitrogen atoms, the nonpolar aromatic groups in A^X^ and C^X^ lack hydrogen donor and acceptor sites. Because perturbation of a DNA duplex structure by the nonpolar group stacking is minimized, it would be possible to evaluate the contributions of polar groups and the polarity of DNA bases to the water binding, which is in progress.

### 3.4. Site-Selective Base Flipping Using the Base Pair-Mimic Nucleosides

Remarkably, even when we did not insert an abasic site in the complementary DNA strand, the deoxyadenosine derivatives adopted the base pair-mimic geometry by intercalating the nonpolar aromatic group in a DNA duplex with minimized disruptions of the overall duplex structure. Strikingly, the Δ*G*_37_° values for forming the DNA duplex containing A^X^ opposite any nucleotide component (A, G, C, and T) were similar to each other and even similar to when the abasic analog was applied. The stacking of the nonpolar aromatic group causes the opposite base to be flipped out of the duplex (Figure 6(b)), and the resultant duplex becomes synonymous in terms of the double helical conformation regardless of the opposite base component, which was verified from CD spectra, fluorescence measurements using the fluorescent base analog 2-aminopurine, and the mobility in polyacrylamide gel [41]. Although the stacking efficiency is largely influenced by the adjacent base pairs, the base flipping conformation was suggested for any type of the closing base pairs.

We also tested the base flipping of an RNA strand. The DNA strand containing the base pair analog was hybridized with a complementary RNA sequence, thereby forming an RNA/DNA hybrid duplex. As predicted from the nearest-neighbor parameters determined for the hybrid duplexes [51], the thermal stability of RNA/DNA duplexes containing a mismatch site differed depending on the type of mismatch pair (the melting temperature *T*_*m*_ of the 11-mer natural duplexes forming A/A, A/G, A/C, and A/U pairs differed by 12.9°C). On the other hand, the duplexes containing A^phe^ or A^naph^ in place of A exhibited almost the same stability, within a 2.0°C difference in the *T*_*m*_ among the duplexes containing A^X^/A, A^X^/G, A^X^/C, or A^X^/U pair. This observation is consistent with the unstacked conformation of the RNA base opposite A^X^. The sugar-phosphate backbone of RNA perturbed due to the unstacking conformation can be preferentially hydrolyzed as a consequence of specific base catalysis at the site adopting the in-line attack arrangement [52, 53]. Indeed, we found highly site-selective cleavage at any ribonucleotide base opposite A^X^ in an RNA/DNA duplex [54]. The RNA-hydrolyzing activity agrees with the base flipping model in which A^X^ forces the opposite base to flip out in an unstacked position (Figure 6(c)). The rate of the RNA hydrolysis was relatively slow comparable to the nonspecific hydrolysis of a single-stranded RNA strand but much faster than those of the unmodified duplexes forming a mismatch pair [55]. Thus, it is likely that A^X^ induces the base flipping of a structurally unconstrained phosphodiester bond as much as ribonucleotides in a single-stranded state. A highly site-selective hydrolysis without base-pairing selectivity has a great advantage for biotechnology and therapeutic uses, thus the RNA cleavage using anoligonucleotide attaching artificial scissors of a metal ion-coordinating group, such as ion macrocycles, cationic amines, imidazole derivatives, and acridine derivatives, has been reported [56, 57]. However, it is usually difficult to restrict the site to be cleaved because of the difficulty in reducing the nonspecific hydrolysis due to conformational flexibility and distortion. The RNA hydrolysis by our base pair analogs is highly site-selective, which arises from minimized disruptions of the double helical structure and the thermal stability.

### 3.5. Dual Conformation Depending on the Interaction Energy

Formation of the base flipping is somewhat surprising from the point view of interaction energy. The flipping conformation is adopted by moving the base from an intrahelical to an extrahelical position. Energetics of the equilibrium between the base stacking conformation and the flipped-out conformation are important to understand the mechanism of the flipping of bases out of a DNA duplex. However, the energy levels of these two conformations are usually largely different, and thus, the base stacking conformation cannot be formed without any cofactor. In nature, the base flipping is seen as an intermediate in the DNA base repair and DNA/RNA base modification pathways, mediated by uracil DNA glycosylase, DNA methyltransferase, and RNA adenosine deaminase, and so forth. These enzymes cause the target base to be flipped out of the duplex, where the energy cost for base flipping is compensated by intercalating the side chain of amino acids and/or forming direct inter actions with the flipped-out base [58–60]. Likewise, the base flipping conformation can be prepared using the porphyryin and pyrene-modified nucleosides that compensate for the energy cost by the intercalation of the large stacking group [20, 21, 61]. Unlike these base analogs with a large aromatic group, our base pair analogs possessing a small aromatic group can provide enough interaction energy to stabilize the base flipping conformation with minimized structural disruptions of the double helical structure. Even when T is located opposite to A^X^, the intercalation energy for the nonpolar group stacking is greater than the interaction energy for base pairing through interbase hydrogen bonds [41, 54].

**Figure 7:**
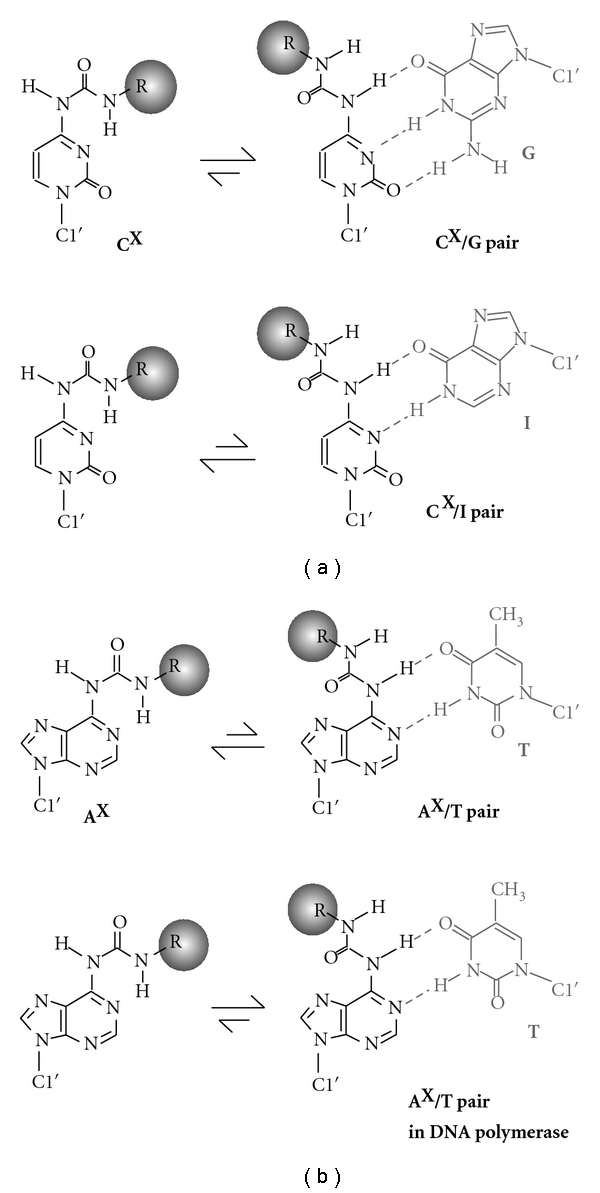
The equilibria between the conformations of the nonpolar group stacking and the base pairing of the deoxycytidine (a) and deoxyadenosine derivatives (b), where R indicates the nonpolar aromatic group of the phenyl or naphthyl group.

It is an interesting finding that the pair-mimic nucleosides can recognize the complementary base under certain conditions, and the conformation changes depending on the interaction energies between the nonpolar group stacking and the base pairing through hydrogen bonds (Figure 7). When the deoxycytidine derivatives were investigated, the thermodynamic stability and the RNA hydrolysis data agreed with the flipping of A, C, T, or U opposite to C^phe^. However, the base pair through hydrogen bonding is formed between C^phe^ and G by orienting the nonpolar aromatic group into the major groove of the duplex, rather than the guanine flipping conformation [55]. This observation is markedly different from the deoxyadenosine derivatives inability to form the base pair with T and U. The importance of the interaction energy in the conformation of C^phe^ was suggested from the studies using inosine (I) lacking the 2-amino group of G. The inosine base opposite C^phe^ was found to be flipped out. This observation clearly demonstrates that the phenyl group stacking overcomes the base pairing of C^phe^/I through two hydrogen bonds but not that of C^phe^/G through three hydrogen bonds (Figure 7(a)). Hence, our base pair analogs can discriminate the bases in accordance with the base pair interaction energy, such as G from I in which the base pair stability with cytosine differed by only about 1 kcal mol^−1^. In a similar mechanism, recognition of a weakened base pair stability is suggested for the DNA damage searching by a DNA repair protein of human *O6*-alkylguanine alkyltransferase [60]. Additionally, we have also found that the triphosphate derivative of A^phe^ can be incorporated opposite T in a DNA template by DNA polymerases (manuscript in preparation), suggesting the conformational change in A^phe^ depending on the molecular environment (Figure 7(b)). It was also found that the equilibrium shift to the nonpolar group stacking conformation was more obvious when the phenyl group was replaced by the naphthyl group. We can conclude that the base pair-mimic nucleosides can potentially adopt a dual conformation in the nonpolar group stacking and the base pairing with a complementary base, depending on their interaction energies.

## 4. Concluding Remarks

This paper describes nucleic acid analogs with the modification and substitution of the purine or pyrimidine base used for the study of nucleic acid interactions. There are also many research studies on the modification at the sugar-phosphate backbone to enhance the hybridization efficiency and to provide greater nuclease resistance for oligonucleotides [62, 63]. For the interaction study, the backbone modification is important to reveal the role of the sugar-phosphate atoms on the cation binding and nucleotide hydration. In comparison to the case of base modifications, molecular design for the backbone modification is more difficult because interactions involved in the backbone atoms are not fully understood. 

Particular base analogs with a nonpolar aromatic group can be applied for the interaction study between DNA and proteins. For example, nonpolar pyrimidine and purine analogs were used to investigate the base pair geometry in the selection of substrate nucleotides by DNA polymerases [64] and the deleterious effects of eliminating a particular base in a DNA duplex on the interaction with topoisomerases [65]. The pyrene-modified nucleotide in a DNA strand was found to be able to restore the catalytic activity of mutant uracil-DNA glycosylases by assisting the target uracil to be flipped out of the DNA duplex [25, 61]. The base pair analogs shown in Figure 3 are the distinguished Watson-Crick base pair analogs that are accommodated to the DNA duplex structure with minimum disruptions of the conformation and the thermal stability, and they can be used for the study of nucleic acid base interactions such as the base stacking, hydration, and DNA-protein interactions. The base flipping conformation prepared using the base pair-mimic nucleosides is useful to cleave a target RNA sequence and allows evaluation of the dynamics and energetics of the base flipping conformation found in the DNA repair and base-modification proteins and in RNA reactions of the mRNA splicing and ribozyme reactions [66, 67]. The base pair-mimic nucleosides also have an outstanding property to adopt the dual conformation responding to the condition, which is useful to investigate base flipping under the equilibrium with base pairing. Therefore, the molecular design using a flexible linker that tethers a modest stacking group to a purine or pyrimidine base is useful to explore base pair analogs useful for studying the biochemical properties of nucleic acid interactions. Modifications at the aromatic hydrocarbon group and the ureido linker may further expand the application of the base pair analogs such as in molecular biology and develop nucleic acid drugs.
